# Lamin A/C Mechanotransduction in Laminopathies

**DOI:** 10.3390/cells9051306

**Published:** 2020-05-24

**Authors:** Francesca Donnaloja, Federica Carnevali, Emanuela Jacchetti, Manuela Teresa Raimondi

**Affiliations:** Department of Chemistry, Materials and Chemical Engineering “G.Natta”, Politecnico di Milano, 20133 Milano, Italy; francesca.donnaloja@polimi.it (F.D.); federica.carnevali@mail.polimi.it (F.C.); manuela.raimondi@polimi.it (M.T.R.)

**Keywords:** lamin A/C, mechanotransduction, laminopathy, gene regulation, lamin partners, Hutchinson Gilford progeria syndrome, Emery-Dreyfuss muscular dystrophy

## Abstract

Mechanotransduction translates forces into biological responses and regulates cell functionalities. It is implicated in several diseases, including laminopathies which are pathologies associated with mutations in lamins and lamin-associated proteins. These pathologies affect muscle, adipose, bone, nerve, and skin cells and range from muscular dystrophies to accelerated aging. Although the exact mechanisms governing laminopathies and gene expression are still not clear, a strong correlation has been found between cell functionality and nuclear behavior. New theories base on the direct effect of external force on the genome, which is indeed sensitive to the force transduced by the nuclear lamina. Nuclear lamina performs two essential functions in mechanotransduction pathway modulating the nuclear stiffness and governing the chromatin remodeling. Indeed, A-type lamin mutation and deregulation has been found to affect the nuclear response, altering several downstream cellular processes such as mitosis, chromatin organization, DNA replication-transcription, and nuclear structural integrity. In this review, we summarize the recent findings on the molecular composition and architecture of the nuclear lamina, its role in healthy cells and disease regulation. We focus on A-type lamins since this protein family is the most involved in mechanotransduction and laminopathies.

## 1. Introduction

Cells perceive different types of stress, ranging from whole-body forces, such as the gravity shear stress of the blood flow, to the microscopic forces induced by interaction with their microenvironment through chemical, electrical, or mechanical cues, such as extracellular matrix stiffness and topography. The mechanotransmission pathway, which transfers the stimuli from the outside of the cell deep into the cell, starts from the extracellular matrix (ECM), goes through several cellular elements, and reaches the cell nucleus eliciting gene expression [1]. The process begins at the interface between the ECM and cells, where the external environment alters the size and orientation of the focal adhesions which, in turn, cause cytoskeletal rearrangements within the cell. The cytoskeleton is a dynamic network of proteins extending from the plasma membrane to the cell nucleus. It is composed of three main components, microtubules, actin filaments, and intermediate filaments, each of them capable of rapid growth or disassembly, according to the cell requirements. The cytoskeleton regulates and mediates vesicular trafficking and cellular signaling, but primarily it confers mechanical stability to the cell and transmits forces from the cytosol to the cell nucleus [2,3,4,5]. At the nuclear envelope, the stimulus is then internalized via the LInker of Nucleoskeleton and Cytoskeleton (LINC) complexes, which mediate the connection between the cytoskeleton and the nuclear lamina [6,7,8]. The nuclear lamina is a thin, but dense, protein meshwork under the inner nuclear membrane, composed of karyoskeletal intermediate filament proteins, named lamins [9,10,11]. The nuclear lamina gives mechanical stability to the nucleus and plays a fundamental role in other cellular functions, such as nuclear localization, cell migration, chromatin organization, epigenetic regulations, and DNA replication and repair [12,13,14,15,16,17]. Interestingly, the nuclear lamina also plays a role in both tissue regeneration as well as in cancer and laminopathies, including forms of cardiomyopathy, muscular dystrophy, lipodystrophy, and aging-related progeria [18,19,20,21,22]. Consequently, understanding the role of lamins in nuclear processes is a key step in revealing the mechanisms behind human diseases and to learn how to treat such diseases. In this review we thus focus on the A-type lamins, the most involved ones in the mechanotransduction process. We then examine the correlation between A-type lamin mutations and laminopathies.

## 2. Lamins

Lamins are the main structural constituents of the nuclear lamina, a mesh-like structure that supports the integrity of the cell nucleus [9,23,24]. In mammalian cells, there are seven lamin isoforms, all of which are a member of the type V intermediate filament (IF)-family. Lamins are classified into two main categories: A-type and B-type. The first group includes the lamins A and C, and the minor isoforms AΔ10 and C2, all resulting from the alternative splicing of the LMNA gene. The second group includes lamin B1, coded by the gene LMNB1, and lamins B2 and B3 from the alternative splicing of the LMNB2 gene. In human somatic cells, the main lamins are A, C, B1, and B2. The isoforms AΔ10 are expressed in tumor cells, and C2 and B3 have been detected only in the germ cells [25,26,27,28,29].

Lamins are dynamic proteins that aggregate and separate according to different stimuli, supplying distinct mechanical properties to the lamina meshwork. To understand how lamins are rearranged, both in vitro and in vivo approaches have been used. In the 1990s, in vitro studies showed that A- and B-type lamins create a mix of heterodimers that together form a half-staggered paracrystalline array. However, these arrays are observed only after the extreme overexpression of lamins, and it seems that they can only be formed in in vitro experiments [30,31]. In fact, in vivo studies performed with 3D-highly-resolved microscopy techniques and by studying lamin knockout and knockdown or their mutations, indicate that A- and B-type lamins form separate networks that overlap below the nuclear membrane and play different roles in cell activities [32,33,34,35,36,37,38]. It is also known that A-type and B-type lamins are differentially expressed according to the cell phenotype [24,32]. In particular, the B-type lamins are constitutively expressed by cells during development, and because they are essential for organogenesis, vital cellular processes cannot take place without them [39,40]. In fact, studies on mice that are lacking lamins B1 and/or B2, reported death shortly after birth with severe defects in neuronal development [41,42,43]. This essential role of B-type lamins in cell viability explains the limited number of mutations observed in LMNB genes and the consequent lower number of associated heritable diseases (see Section 5) [44,45]. Nevertheless, lamin B1 may be involved in cellular senescence, due to its reported loss in senescent cells [46,47,48]. Instead, A-type lamins are predominantly expressed in the most differentiated cells (except for certain cells of the hematopoietic system and mammalian germ cells), and play a pivotal role during their differentiation [49,50,51]. Indeed, while the nuclei in embryonic stem cells are extremely soft, with a low expression of A-type lamins, the nuclear stiffness and the number of A-type lamins increase during cell differentiation and embryonic development. Unlike B-type lamins, a lack of A-type lamins is not incompatible with cellular life, as highlighted by the various mutations of A-type proteins causing the heritable diseases known as laminopathies. These kinds of disorders manifest themselves predominantly in mesenchymal tissues and include forms of lipodystrophy, muscular dystrophy, cardiomyopathy, and aging-related progeria [14].

**Figure 1:**
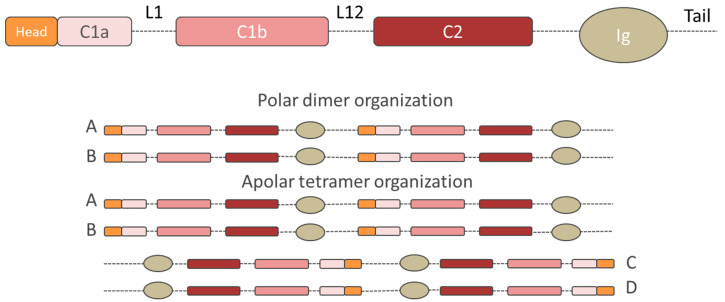
The general structure of lamins. From top to bottom: single chain structure, dimer organization and tetramer structure. The structural organization of human lamins consists of head, central rod, and C-terminal tail containing the globular Ig-like domains. Dotted lines represent unstructured regions. The central rod domain is divided into the three coiled coil domains C1a, C1b, and C2, separated by the flexible linkers L1 and L12. Two single chains associate with their coiled coil domains to form parallel (polar) homodimers, which bind other identical homodimers via head-to-tail interactions thus forming a polar structure (chain A and chain B). In line with other IF structures, the homodimers interact laterally with other identical homodimers arranged in an antiparallel way. The resulting structure is an antiparallel (apolar) tetrameric filament (chain A, chain B, chain C, and chain D).

**Figure 2:**
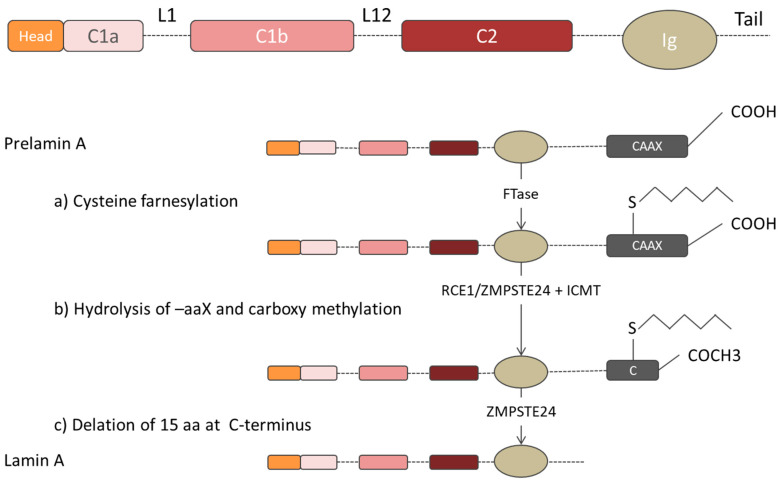
Prelamin A post-translational modifications during the maturation phase. (**a**) Cysteine farnesylation of prelamin A at the -CAAX box by Farnesyltransferase enzyme (FTase). (**b**) Hydrolysis of –aaX motif by either RCE1 or ZMPSTE24 enzyme and C-terminal methylation at terminal cysteine via ICMT. (**c**) Mature lamin A structure after the cleavage of the last 15 amino acids via ZMPSTE24 enzyme.

All the lamins are characterized by similar amino-acid sequences and molecular structure, which differ in terms of molecular weights: *LMNA*-encoded lamins A, AD10, C correspond to 70, 66, and 61 kDa, respectively, and B-type lamin isoforms correspond to 67/68 kDa. All these proteins consist of three structural domains: A N-terminal head, a central coiled-coil region, and a large globular carboxyl-terminal tail (indicated in orange, pink-red, and with dotted line, respectively, in Figure 1) [25]. The amino-terminal head consists of an unstructured variable sized region. Instead, the central α-helical rod domain is highly structured and spans almost half of the entire protein (about 350 residues) and it is arranged in at least three α-helical segments (coil 1A, coil 1B, and coil 2), characterized by typical coiled-coil heptad-repeat pattern and connected by short intermediate sub-domains termed L1 and L12 [23]. The carboxyl-terminal tail domain includes an immunoglobulin-like (Ig-) fold domain, the nuclear localization signal (NLS) for lamin transport into the cell nucleus, the chromatin binding site and, except for lamin C, a cys-aliphatic-aliphatic-any residue box (CAAX) [23,52,53]. The Ig-fold domain mainly consists of two β-sheets, made up of five and four β-strands, respectively, connected by a short loop forming a compact β-sandwich [54]. Lamin C is translated as a mature protein [55] and lacks 98 C-terminal amino acids present in pre-lamin A, including the −CAAX box [56,57]. Instead, B-type lamin proteins and lamin A protein, are initially translated in the prelamin form, characterized by specific -CAAX box (CSIM, CAIM, and CYLM for lamin A, B1, and B2, respectively), tied to the end of the C-terminal domain [58,59,60,61]. The prelamin forms are subjected to post-translational modifications to reach the respective mature forms [55,60,62]. The post-translational modification begins with the farnesylation of the C-terminal cysteine, via the farnesyltransferase enzyme (Ftase) that adds a 15-carbon farnesyl isoprenoid to the carboxyl terminal cysteine [59,63]. The three residues (aaX) are then cleaved off by the prenyl-CaaX-specific endoprotease: while the RAS converting enzyme 1 (Rce1), also known as Farnesylated proteins-converting enzyme 2 (FACE-2), acts on the B-type lamins, both Rce1 and zinc metalloproteinase Ste24 homologue (Zmpste24), also known as Farnesylated proteins-converting enzyme 1 (FACE-1), can be responsible for the cleavage of prelamin A [64,65]. Then the carboxyl terminal cysteine is carboxymethylated by the isoprenylcysteine carboxyl methyltransferase (ICMT). While the mature B-type lamins permanently maintain this form, which facilitates their stable localization at the nuclear envelope, pre-lamin A undergoes an additional step [66]. Indeed, pre-lamin A is subjected to Zmpste24-dependent cleavage between Tyr-646 and Leu-647, resulting in the removal of the last 15 amino acids, releasing the mature lamin A [67,68,69,70]. Mature lamin A terminates at Tyrosine 646 (Y646) and has 18 amino acids less than its precursor [52]. Figure 2 shows the lamin A post-translational modifications during maturation. The B-type lamin permanent farnesyl moiety plays a relevant role in B-type lamins localization in cell cycle. During the cell cycle, lamins show several other types of post-translational modifications such as sumoylation, ubiquitylation, and acetylation, which are involved in lamin turnover and lamin translocation to the cell nucleus [71,72,73]. The only easily-reversible modification is phosphorylation, which enables a rapid alternation between the activation/inactivation protein states [58]. This process governs lamin functions, such as its targeting to specific locations or the immobilization of interacting protein complexes. Above all, it regulates lamin A/C solubility and controls the lamina meshwork formation [58,74,75]. Phosphorylation is a modification required at the onset of mitosis and during interphase, and involves several protein kinases (e.g., Cdk1, Cdk4, Cdk6) [58,68,76,77,78,79]. During mitosis, the nuclear envelope breaks down, and the nuclear lamina dismembers; while the phosphorylated A-type lamins are solubilized in the cytoplasm and the nucleoplasm, B-type lamins maintain close associations with the nuclear envelope due to its farnesyl moiety, as shown by Moir et al. by using confocal fluorescence imaging of PAM cells expressing green fluorescent protein (GFP)–laminA and (GFP)–laminB fusion proteins. During G1 phase, the phosphorylation process guarantees lamin turnover, during which non-essential A-type lamins are degraded and the others are reassembled into the nuclear lamina [38,74]. However, irrespective of the cell cycle phase, phosphorylation modulates the solubility of the intermediate filament proteins driving lamina assembly/disassembly, as a mechanosensing response to external mechanical stimuli [80,81]. The tension-induced changes in A-type lamins, suppressing the affinity to the kinases, explain the high lamin turnover process [82]. This highly transient attitude of A-type lamins is shown by dynamic studies on GFP-lamin A proteins. For example, Bronshtein at al. used continuous photobleaching (CP) experiments to extract the ratio of free-to-bound lamin A in the cell nucleus. They observed that the fraction of freely diffusing lamin A in the nucleoplasm was ∼60%, meaning that 40% of lamin A was bound immobile [83].

**Figure 3:**
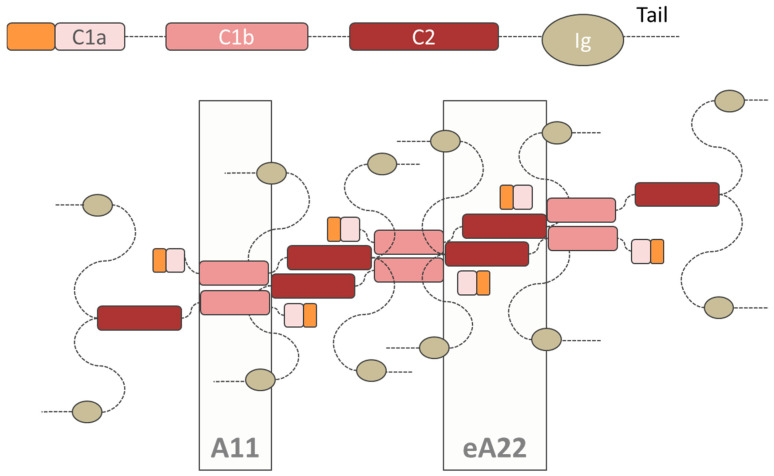
Tetrameric structure of the lamins proposed by Ahn et al. The structure of the polar dimers is reported as a single chain. In this innovative model, both the interactions A11 (C1b domain interaction) and eA22 (C2 domain interaction) are satisfied, while still maintaining the antiparallel layout of the dimers.

Regardless their differences in terms of stability at the nuclear periphery, all lamin types arrange in a similar way to form the lamina network. In particular, the process starts with the dimer assembly, which is the fundamental soluble unit of the lamina; lamins dimerize using their α-helical heptad repeat periodicity, where amino acids in positions “a” and “d” contain preferentially hydrophobic residues, while repeats “e” and “g” are charged residues. A hydrophobic seam runs along the α helix axis, acting as a dimerization interface between the two parallel-oriented proteins. The two α helixes are coiled around each other, thus resulting in lamin dimerization. The dimers then aggregate head-to-tail, forming small polymer filaments. Although these filaments are then rearranged into a regular woven meshwork pattern, their assembly mechanism and the 3D structure are not well understood [9,23]. In fact, because of its intrinsic flexibility the lamin structure has been determined only by low-resolution electron microscopy. The high-resolution X-ray technique only solved the limited and short fragments listed in Table 1 and Table 2 [84]. Recent studies support the hypothesis that lamin filament tetramers assemble in cross-sections. However, while in vitro tests have shown that lamins are organized in 10 nm thick-filaments [23,85], cryo-electron tomography tests have revealed that lamins assemble into thinner fibers (about 3.5 nm thick), with a larger globular zone only at the Ig-fold domain [9]. In this context, Ahn et al. proposed a new model describing the tetrameric arrangement of lamins, which is consistent with the 3.5 nm thickness restriction and the two proposed binding regions, named eA22 and A11 [23,86]. To facilitate the comprehension of the model, Figure 3 reports the polar dimer structure as a single chain. Further studies are required to validate the proposed model.

Cells can modulate the nuclear biophysical properties by changing the phosphorylation level of the lamins, thereby affecting both the structural lamina conformation and stiffness [87]. Cells also react in response to the extracellular stimuli by modulating the lamins expression levels, thus highlighting the major role of the A-type lamins in nuclear stiffness [8,16]. In fact, under stress, A-type lamin-deficient cells exhibited reduced nuclear stiffness, misshapen nuclei, and decreased cell viability; whereas lamin B1-knockdown cells had no effect on the nuclear stiffness, but increased nuclear blebbing and altered nuclear integrity, which are correlated to scarcity in cell survival [11,16]. There are also mild variations in the lamina levels that affect fundamental cell processes, such as the cells ability to differentiate. For instance, Discher et al. demonstrated that A-type lamin expression increases about 30-fold from soft (like brain) to stiff (bone) tissue, while B-type expression differed less than 3-fold. In accordance with their constitutive expression, B-type lamins are not sensitive to mechanochemical regulation [82,88,89]. While it is possible to consider the amount of B-type lamins as being constant, mass spectrometry has shown an A-type lamin expression level proportional to E^0.7^, where E represents the tissue microelasticity. The coefficient 0.7 is calculated as a geometric mean between A and C lamins, even though the specific scaling factors are 1.0 and 0.5, respectively. It is therefore evident that B-type lamins dominate in the soft tissue, while A-type lamins play a key role in guiding cell functions in stiff tissues [82,89], in fact its expression level correlates with cell phenotype [50,90,91]. For example, in mesenchymal stem cells (MSCs), A-type lamins are almost absent during stemness maintenance but increase during differentiation; low levels of lamins have been found during the adipogenic differentiation but increase during the differentiation to chondrogenic and osteogenic phenotypes as shown in [92] by single cell immunofluorescence and by immunoblot experiments. This trend has been confirmed by in vivo tests using histological and micro computed tomography measurements of mouse femurs. The results showed that in mice lacking A-type lamins, bone loss occurs due to a preferential MSC differentiation toward adipocytes and not osteoblasts. Moreover, Li et al. determined the femoral bone loss observing a significant decrease in trabecular number, an increase in trabecular separation, and a much lower cortical thickness. These data suggested that the MSC preferential differentiation toward adipocytes is a key feature in age-related bone loss. [93].

**Table 1:** Wild type human lamin A/C fragments obtained by X-ray.

PDB Code	Segment	X-ray Resolution	a.a.	Chain
1X8Y [94]	Coil 2B	2.2 Å	305–389	Single chain (A)
3V5B [95]	Coil 2B	3 Å	313–386	Single chain (A)
1IFR [54]	Globular domain	1.4 Å	436–552	Single chain (A)
2XV5 [96]	Coil 2B	2.4 Å	328–398	Dimer (A + B)
6SNZ [86]	Coil 1B	2.6 Å	65–222	Tetramer (A + B + C + D)
6JLB [23]	Head-coil 2	3.2 Å	1–300	Tetramer (A + B + C + D)

The table lists the fragments PDB codes, the corresponding domains, the X-ray resolutions achieved, the amino acids involved, and the name of the solved chains of the A-type lamins.

**Table 2:** Wild type human lamin B1 fragments obtained by X-ray.

PDB Code	Segment	X-ray Resolution	a.a.	Chain
3UMN [97]	Globular domain	2 Å	428–550	Trimer (A + B + C)
3TYY [97]	Coil 2B	2.4 Å	311–388	Dimer (A + B)

The table lists the fragments PDB codes, the corresponding domains, the X-ray resolutions achieved, the amino acids involved and the name of the solved chains of the lamin B1.

## 3. Lamin Binding Partners

**Figure 4:**
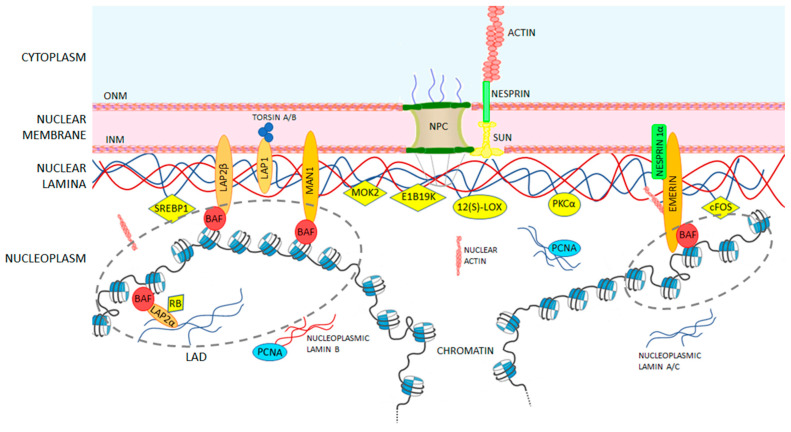
A-type lamin-binding partners at the nuclear envelope (NE) level. Lamina underlines the nuclear membrane and consists of A-type lamins (in blue) and B-type lamins (in red). Proteins interacting with A-and B-types lamins are schematized and their localization into the cell nucleus. The proteins interacting with lamins at the level of the nuclear envelope are thought to have mainly a mechanical and structural role; proteins bridging directly lamins and chromatin play a key role in reinforcing the nucleoskeleton and in mechanical regulation of gene transcription; others regulate cell signaling.

**Figure 5:**
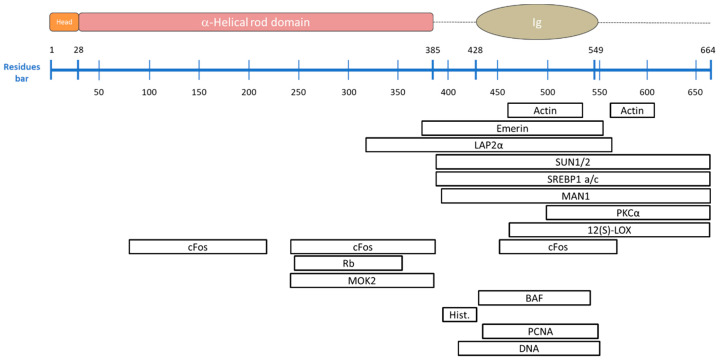
A-type lamin-binding proteins and their known binding site. (**Top**) a scheme of the pre-lamin A structural domains, schematized in the N-terminal head, the central coiled-coil region (including the coil 1A, coil 1B and coil 2 shown in the previous figures), and the C-terminal tail including the Ig-fold domain. The residues bar indicates the positions of the respective amino acids. The amino acids after residue 646 are normally removed by proteolytic cleavage to generate mature lamin A. Lamin C is identical to lamin A till residue 566 and contains 6 lamin C-specific amino acids at its C-terminus. (**Bottom**) List of A-types lamin binding proteins and their relating binding sites.

Lamins are located in the cell nucleus. Once reticulated in a mesh forming the lamina, they localize close to the nuclear envelope (NE), where they interact with several other proteins. Although most of the NE-associated proteins directly bind to A- and/or B-type lamins, others need mediators [68,98]. A-type lamins anchor proteins involved in cell signaling and chromatin remodeling. Lamins are thus involved in a wide range of nuclear functions, such as cell proliferation, cell migration, genome organization, and DNA repair, as well as serious diseases, such as laminopathies [99,100,101,102]. Lamin binding partners are divided in the three main groups. The architectural partners connect lamins to the nuclear envelope, chromatin, or other subnuclear structures, thus providing nuclear mechanical support. The signaling partners that are involved in the regulation of many cellular functions, such as cell differentiation and homeostasis. The chromatin and gene-regulatory partners that regulate chromatin localization and gene expression. Table 3 summarizes the main binding partners of lamin A/C and their interaction domains. Figure 4 and Figure 5 represent protein localization and protein-lamin A/C connections.

## 4. Lamin A/C Roles in Cell Mechanotransduction

**Figure 6:**
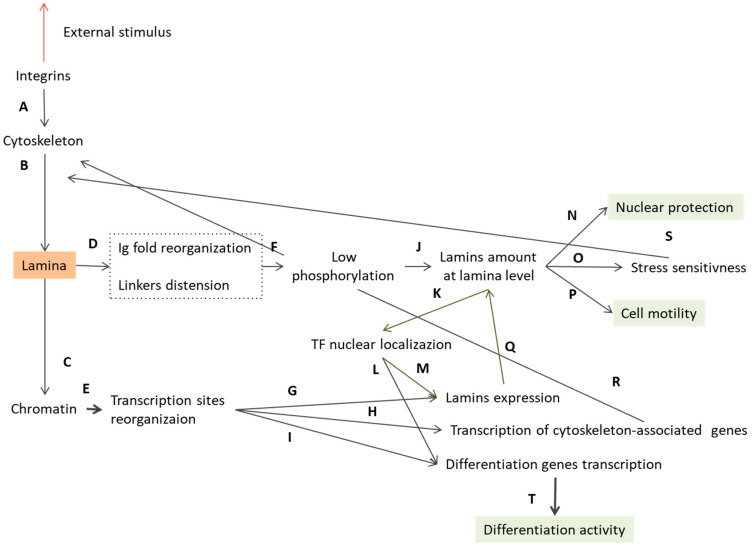
The role of lamina in mechanotransduction. Starting from the extracellular matrix, integrins transmit the external impulse to the cytoskeleton (**A**), which then transfers the stimulus to the lamina structure (**B**). The lamins first act as a mechanosensor and rearrange their molecular structures (**D**), hiding the sites for phosphorylation (**F**). The inhibited phosphorylation activity increases the amount of lamins at the nuclear envelope with a consequent increase in lamina stiffness (**J**). At this level, the lamina mesh guarantees the nuclear protection (**N**) and higher stress sensitivity (**O**), which impact on the rearrangement of the lamina (**S**) and affect cell motility (**P**). Lamina rearrangement is correlated to the higher nuclear localization of some transcription factors (**K**), whose activity is also enhanced by the alteration in chromatin structure (**C**). The rearrangement of the lamina leads to a reorganization of the chromatin (**C**), thus altering the sites available to the transcription factors (**E**) for lamins (**G**), cytoskeleton (**H**), and differentiation genes (**I**). The reorganization of the chromatin sites and the higher nuclear availability of transcription factors alter the transcription of the lamins (**M**,**G**) which thus provides feedback on the lamina stiffness (**Q**). The chromatin reorganization also acts on the transcription of the cytoskeleton components (**H**), which in turn affects the force transmission to the lamina at the upstream level (**R**). To conclude, the chromatin reorganization affects the differentiation gene transcription (**I**), which, combined with the increased transcription factor nuclear localization (**L**), act on cell differentiation activity (**T**). Orange is used to highlight the key position of the lamina at the crossroads of the mechanotransduction pathway, and green highlights all the revealed downstream effects of the lamina reorganization. Green arrows represent the lamins feedback loop.

**Figure 7:**
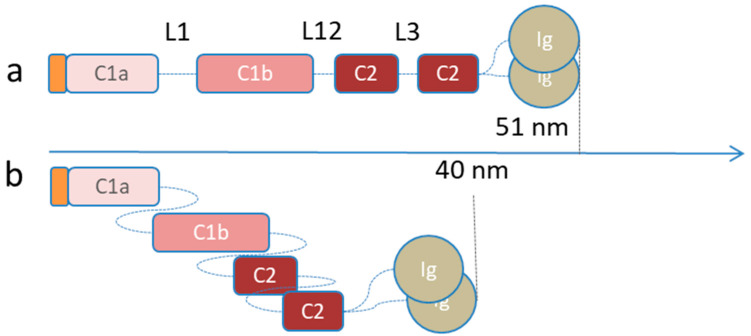
Stable conformations of the lamin dimers. (**a**) Lamin dimers in semi-relaxed configuration (total length about 50 nm). (**b**) The rod of lamin dimers can compress via three sequential tandem staggering of linkers L1, L12, and L3 (total length about 40 nm) for electrostatic interactions.

**Figure 8:**
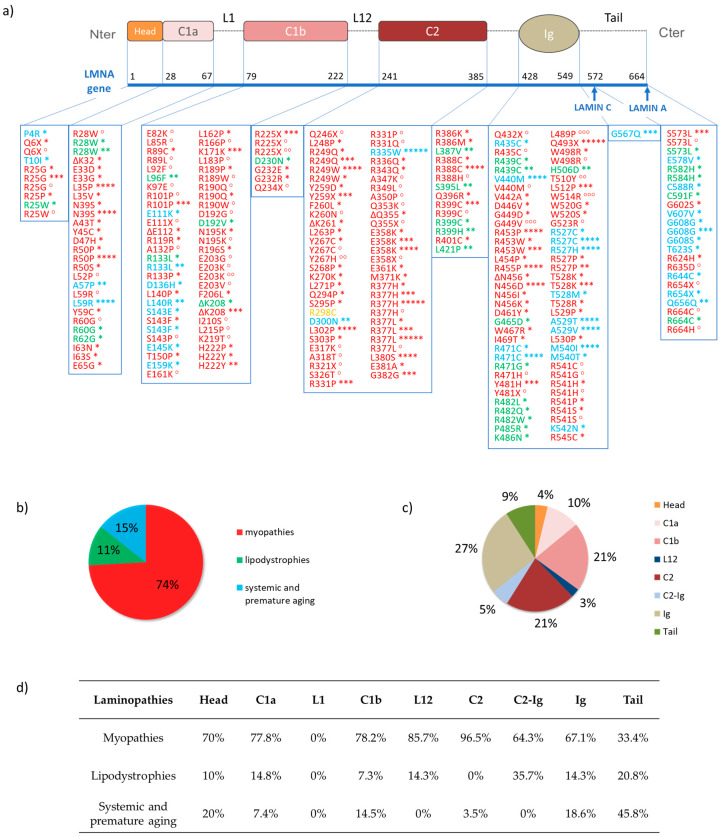
The single-point mutations of the *LMNA* gene. (**a**) List of *LMNA* gene mutations graphically associated with distinct lamin domains. Red indicates the gene mutations related to the following myopathies: EDMD2 (*), EDMD3 (**) LGMD1B (***), CMD (****), AS-SMA (*****), CDM1A (°) and DCM-CD (°°); mutations associated with various uncategorized phenotypes of muscular dystrophy, as reported by Dialynas et al. [182] are also reported in red (°°°). In green, those regarding lipodystrophies: FPLD2 (*) and MS (**). In yellow, the mutations causing the CMT2B1 neuropathy. Finally, blue indicates the gene mutations relative to systemic and premature aging disease: HGPS (*), WRN (**), RD (***), MADA (****), HHS (*****). (**b**) The percentages for each group of laminopathies. Almost 74% of the single-point mutations cause myopathies. Premature aging and lipodystrophy are 15% and 11%, respectively. Only one mutation has been associated with neuropathy. (**c**) The percentages for each lamin domain. Ig-fold domain, C2 and C1b involve most of the known mutations, representing 27%, 21%, and 21% of the entire set of mutations, respectively. They are followed by C1a (10%), tail (9%), the domain between C2 and Ig-fold (C2-Ig) (5%), the head (4%), and finally L12 (3%). No mutations have been correlated with L1. (**d**) Table collecting the percentages related to the mutations classified according to both the domain and the group of laminopathies.

The overall cellular response is activated by the mechanotransduction pathway, where the A-type lamins play a key role [14,137,138]. Mechanotransduction is the cell capacity to transduce the mechanical signals into a biological response by acting on several cell functions, such as cell growth or differentiation and disease progression. In this context, the lamina first acts as a mechanosensor (Figure 6A,B), so that it can sense the external stimuli, and then as a mechanotransducer, converting the information into other cellular responses. Because of mechanical external stimuli, lamins are rearranged at the molecular level (Figure 6D) thus affecting the lamina 3D mesh. Some researchers have demonstrated that shear stress applied to isolated nuclei causes the lamin A immunoglobulin domain to unfold (Figure 6D). They identified the 522Cys amino acid as a stress-sensitive site, which opens the domain, thereby altering the interactions of the lamins [82]. In line with this theory, other researchers observed an impaired Ig-fold domain opening in pathologic cells [139,140]. This theory is supported by mutations affecting the Ig-fold domain that covers 27% of the total amount of the known laminopathic-related mutations. A more detailed analysis is given in Section 5 and reported in Figure 8. In the same context, Makarov et al. proposed another lamin molecular structure rearrangement according to different stress conditions (Figure 6D). They presented the flexible linkers L1, L12 (shown in Figure 1), the additional putative linker, named L3, and the dimer head-tail interaction (Figure 2), all as springs involved in the lamina stretch and compaction properties. In in vitro conditions, Makarov et al. proposed two possible dimer states named “semi-relaxed” and “compression configuration”, compatible with the dimensions (40–50 nm) obtained from rotary metal shadowing EM (Figure 7). Electrostatic interaction drives the lamins coiled coils sliding to each other favoring the lamin contraction. Moreover, the lamin tail domain plays a relevant function in lamins arrangement because of its peculiar positive charge right after the rod domain (aa 403–407). This positive charge favors electrostatic interactions, crucial for lamin assembly. In particular, at the tetrameric level the positively charged tail allows the crosslinking to the negatively charged coil 1A, L1 and coil 1B regions. This flexible connection with the rod at the tetrameric interface would allow further compression of the entire structure in rest condition. Instead, under stress condition, the interactions of the lamins are broken, and all the flexible regions of the lamins are stretched, thereby extending the rod domain via proximal re-organization of electrostatic and polar interactions [84]. Although the stress-induced rearrangement of the lamins is not clear, the molecular changes in the lamins alter their interaction with other molecules, affecting the entire structure of the lamina meshwork (Figure 6F) [141]. In addition to the stress-related changes in terms of lamin rearrangement, the total amount of lamins is also sensitive to mechanical stimuli. Stem cell studies have revealed how the physical properties of the environment modulate the expression of A-type lamins, irrespectively of the initial lamin levels [82,142]. For instance, Swift et al. demonstrated that glioblastoma tumor cells grown in subcutaneous flank sites, returned a lamin-A:B ratio which was 1.5-fold higher than the same cells implanted in a mice brain. If B-type lamin expression can be considered as being almost the same in the two implantations, the increase in A-type lamins in the flank condition validates their compliance to external stimuli [82]. In relation to the stress-dependent mechanisms modulating the expression of A-type lamins, there are several theories ranging from the lamin A-phosphorylation mechanism to the feedback-controlled transcription regulation. For instance, Buxboim et al. found an increased level of phosphorylated soluble lamins in cells cultured on a soft matrix. They suggested that there is a stress-mediated A-type lamin localization balance between the nuclear lamina and the nucleoplasm, which is controlled by phosphorylation [80]. The inverse correlation between phosphorylation and matrix stiffness has also been reported. For example, cells cultured on soft tissue (0.3 kPa) showed a wrinkled nuclear envelope and higher phosphorylation activity, which promote the disassembly and turnover of lamins. On the other hand, cells on a stiffer matrix (40 kPa) are characterized by flattened, smooth, and stiffer nuclei, with fewer phosphorylated lamins at amino acid Ser22, which is one of the best characterized phosphorylation sites in lamin A/C [80,82]. The higher amount of lamins associated with the lamina mesh, in turn increases the stiffness of the matrix (Figure 6J). The increase in lamina stiffness may also be due to an increase in the nuclear translocation of some transcription factors, such as the lamin-promoting transcription factor retinoic acid receptor g (RARG), myocardin-related transcription factor (MRTF), serum response factors (SRF), and Yes-associated protein 1 (YAP1), which are proteins involved in epigenetic cell regulation (Figure 6K) [82,143,144,145]. It has also been demonstrated that A-type lamin knockdown correlates with inhibited RNA polymerase II transcription and with the suppression of the proteins involved in regulating cytoskeletal-related gene activation (Figure 6H) [146]. As a result, the modulated cytoskeletal transcription provides feedback on the external force transmission to the actin-associated lamina (Figure 6R) [25]. Although the lamina role in the transcription factor translocation is not clear, two hypotheses have been developed. They both focus on the nuclear pore complex (NPC), a multiprotein structure which spans the nuclear envelope and is the only gate for the nuclear transport of macromolecules [8,74,147,148]. The first theory suggests a binding mechanism between the nuclear pore and the lamina, which, when cells are subjected to external stimuli, activates the NPC opening, influencing the influx of transcription factors [6,149,150]. The nuclear pore opening has already been experimentally validated [149,150]. The second theory proposes the involvement of lamina in NPC formation and localization. In fact, A-type lamin appears to play an essential regulatory role in pore distribution across the nuclear envelope and in the formation of “pore-free islands” zones without the presence of NCPs. These regions are rich in A-type lamins and emerin, but they lack B-type lamins. This emphasizes the importance of A-type (rather than B-type) lamins in mechanotransduction [74,151]. Other studies have added new insights into the relationship between A-type lamins and nuclear pores: In cells lacking lamin A, NPCs are found in lamin C-rich regions and in lamin C-deficient cells, they are distant from lamin A. These observations suggest that nuclear pores are linked directly to lamin C, rather than to lamin A, because the lamin C Ig-fold domain shows less steric hindrance compared to the same domain of lamin A [24]. To further investigate the role of lamins in this regulatory mechanism, Maeshima et al. performed studies by silencing proteins. In emerin-reduced cells, the pore-free islands were not affected, while the knockdown of A-type lamin led to a dramatic dispersion of the islands and emerin release into the cytoplasm [74]. Mimura et al. demonstrated that an ectopic expression of A-type lamins induces the formation of pore-free islands, underlying the direct role of A-type lamins in pore dynamics and in keeping emerin proteins in the nuclear envelope [151]. There is also a zone rich in condensed heterochromatin in pore-free islands. Since heterochromatin houses silent genes, this result suggests that the A-type lamins might rearrange the NPC distribution in order to promote molecular transport only in NE regions where heterochromatin is located [74,152]. Aside from the exact mechanism regulating the transcription factor nuclear translocation, which remains unclear, some researchers have investigated the effect of the nuclear transcription factor localization in terms of cell activities. They observed that the lamina network modulation is based on A-type lamin transcription feedback regulation (green arrows in Figure 6), where the LMNA-mRNA expression level depends on the existing amount of *LMNA*-related proteins, which is regulated by a tension-dependent degradation rate. It seems that the high level of A-type lamins induced by the external stimuli via inhibited phosphorylation (Figure 6A,B,D,F,J), facilitates the translocation of RARG into the nucleus (Figure 6K), which then promotes the lamin A transcription (Figure 6M), thereby increasing the number of peripheral lamins (Figure 6Q) [82,144]. The same researchers also demonstrated that a low expression level of lamin A leads to the highest cytoplasmic level of RARG, with the consequent lowest promotion of *LMNA*. The lamina feedback regulation model describes how the stress-regulated protein turnover ensures steady-state A-type lamin level that changes according to the matrix stiffness. In particular the lamin level results proportional to (tissue stiffness)^0.7^ [80,82].

Although the feedback loop of lamins has not yet been completely validated, all the results on stress-related changes in A-type lamin expression, highlight the lamina role in protecting the nuclear integrity (Figure 6N) and in regulating cell functionality (i.e., cell proliferation, migration and differentiation) (Figure 6P,Q) [10,11,82,153]. In fact, from a mechanical point of view, the lamina meshwork behaves like a viscoelastic structure. During micropipette aspiration tests, the nuclear compliance has been calculated as a combination of time, aspiration pressure, and lamin expression level. While B-type lamins give elasticity to the nucleus thereby facilitating its return to the original shape, A-type lamins appear to behave like a viscous fluid that impedes nuclear deformation. To better understand the nuclear compliance, the researchers calculated the elongation response time (τ = viscosity/elasticity). This parameter is an index of time required for nucleus rearrangement after a stress stimulus, indicating the time that the viscous component needs to dissipate the stored energy after a rapid stretching. It is a function of the lamin A:B concentration ratio and the elongation response time resulted in ~2.5 (laminA:B). The τ variation consistent with the specific amount of A-type lamins (almost 10,000-fold across various glial, epithelial, and mesenchymal cell types with or without knockdown or over expression of lamin-A) impedes rapid nuclear distension on stiff substrates, acting as a shock absorber for nuclear integrity [82,154]. However, micropipette aspiration tests measure the nuclear mechanical response to a large (>100%) non-physiological and sudden extensional stress applied to a small area, and do not separate the chromatin and lamin contributions to the whole nuclear response [155]. To circumvent these effects, a tailored micromanipulation force measurement technique has been developed to perform nuclear force-extension measurements on isolated cell nuclei, gradually stretched at a physiological rate to physiological strains. The authors of this technique tested the differential contribution of nuclear components. They found that A-type lamins are major determinants in nuclear strain stiffening at large extension (>3 µm), while the chromatin is responsive above all to small extensions (<3 µm). Cells subjected to small extensions showed the chromatin modulation of the nuclear stiffness via euchromatin/heterochromatin levels, returning a linear force response. The nuclear spring constant increases to 2.5-fold, if subjected to long extensions. In line with previous studies, the authors suggested that the nuclear stiffening is largely due to both the geometry and stoichiometric ratio of the lamin A to lamin B increase in cells on a stiff substrate [155].

In addition to the cellular mechanical support, lamins cover a multitude of other functions, related to chromatin organization, gene regulation, and cell fate determination. The substrate stiffness correlates to the amount of A-type lamins and the phenotype of the cells [154,156,157]. For example, Heo et al. showed that compliant external stimuli favor the MSC differentiation into adipocytes, induced by scarce cell focal adhesions and therefore, soft nuclei and inhibited A-type lamin production [157]. Other researchers, instead, demonstrated that more rigid substrates (around 10 kPa) induce cell spreading and a higher A-type lamin expression with consequent cells differentiation into myocytes [82,158]. Finally, Discher et al. observed that high substrate stiffness (in 100 kPa magnitude order) induces high expression levels of A-type lamins, high lamina network organization, and MSC differentiation to osteogenic phenotype [4,82]. Other researchers have investigated the phenomenon from a different point of view, looking at the fundamental factors for cellular phenotype maintenance during in vitro expansion. For example, Raimondi et al. observed the long-term maintenance of the stemness in MSC grown in a three-dimensional scaffold that was able to maintain the cell nucleus in a roundish configuration, weakly subjected to external forces [159]. The cellular phenotype expression, similarly to the modulation of all the other cellular functions, can be explained by considering the lamin interaction with the chromatin either directly or through histones and other lamin-associated proteins (Figure 6C). As demonstrated by biochemical assays and electron microscopy, these interactions occur both at the nuclear edge and within the nucleoplasm, rearranging the chromatins and altering the accessibility of transcription factors to gene- binding sites (Figure 6E) with a consequent change in gene expression (Figure 6I,Q) [10,160,161,162,163]. For example, lamina-associated domains (LADs) play a fundamental role determining the overall spatial organization of genomes and form repressive chromatin environments with low gene-expression levels [164,165]. They are dynamic domains characterized by spatial positioning that varies according to cell-type specific gene expression activities [161]. In mammalian cells, LADs cover about 30–40% of the genome and mainly bind to the heterochromatic histone markers, such as H3K9me2, H3K9me3 and H3K27me3, which are localized in proximity to the nuclear envelope. However, they can also be observed in the nucleoplasm. For example, after mitosis, LADs are seemingly randomly redistributed throughout the nucleus of the daughter cells, with only a subset displaced at the nuclear periphery [166]. Fluorescence microscopy has revealed that nucleoplasmic lamin A binds the LADs, holding them mainly close to the nucleoli. When this occurs, the chromatins are rearranged with a consequent increase in transcriptional activity [31,166,167,168]. The interaction between LADs and lamins takes place in all cellular phases, and their balance varies depending on the cellular state. In support of this scenario, some researchers have studied the localization and the conformation of the chromosomal territories, correlating them with the state of pluripotent stem cells. For example, biochemical assay in combination with high spatial resolution fluorescence microscopy measurements showed that pluripotent stem cells are characterized by a low level of lamin A and heterochromatin (H3K9me3) expression as well as high levels of euchromatin (H3K4me3) expression. In these cells the chromatin is homogeneously diffused, thus facilitating the link with the transcription factors, while in adult cells or during the cellular differentiation phase, dense areas of heterochromatin appear to inhibit the diffusion of transcription factors and binding to the DNA [169,170]. Discher’s studies on MSCs seem to support this model. In fact, at the point where MSCs grow on high-hardness substrates (in the order of 100 kPa), there was an approximately 30-fold increase in lamin A expression. This massive increase in lamin A sequesters the LADs, influences their localization and their bond with chromatin, leading to a remodeling of the DNA packaging, and therefore, regulating the gene transcription activity and the cell phenotype (Figure 6T) [82].

In summary external mechanical stimuli-induced lamina structural modulations mediate several cellular functions, such as viability, proliferation, migration, and differentiation. Lamina is therefore a key player in cell development, wound healing, hematopoiesis, and it is also involved in human diseases such as cancer and laminopathies.

## 5. Lamin A/C Mechanotransduction in Laminopathies

Laminopathies are heritable human diseases associated with several mutations in lamins and lamins-associated proteins [14,22,137]. A total of 90% of known laminopathies relate to the *LMNA* gene (Figure 8), and only two diseases are reported to be linked to mutations in *LMNB1* or *LMNB2* genes: the autosomal-dominant leukodystrophy and Barraquer-Simons syndrome, respectively [44,45,171]. Laminopathies are usually classified into four groups, according to both the number and the types of the affected tissues, as reported by UMD-LMNA, the universal mutations database (available at www.umd.be/LMNA/). The first group represents the myopathies affecting both the skeletal and the cardiac muscle. This disease class includes Emery-Dreifuss muscular dystrophy (EDMD), Limb-Girdle muscular dystrophy type 1B (LGMD1B), autosomal dominant spinal muscular dystrophy (AD-SMA), congenital muscular dystrophy (CMD), and dilated cardiomyopathy (CMD1A) [172,173,174,175]. The second group includes lipodystrophy diseases that affect the adipose tissue with consequences on metabolic pathway malfunction. The main pathologies are Dunnigan-type familial partial lipodystrophy (FPLD2), and the metabolic syndrome (MS) [176,177]. The third group represents neuropathies, which affect the neural tissue such as Charcot-Marie-Tooth disease (CMT2B1) presenting a damaged peripheral neuronal system [178]. Lastly, the laminopathies belonging to the fourth group are multisystemic disorders, such as premature aging syndromes, mandibuloacral dysplasia and Werner syndrome. Of these, the most studied subtypes are the Hutchinson-Gilford progeria syndrome (HPGS), the atypical Werner syndrome (WRN) and the mandibuloacral dysplasia with lipodystrophy of type A (MADA) [179,180,181]. Most of the laminopathies are autosomal-dominant diseases caused by single point mutations. Quantitative analyses appear to indicate that 74% of the known mutations cause myopathies, whereas 11% and 15% are associated with lipodystrophy and premature aging, respectively. These mutations mainly occur in the Ig-fold, C2 and C1b domains, which involve 27%, 21%, and 21%, respectively, of the entire mutations set (Figure 8). Table 4 reports the four families of laminopathies, their specific diseases and the mutated genes involved. Figure 8 gives the specific mutations of the *LMNA* gene for each pathology along with some statistics correlating pathologies and gene mutation.

The pathological mechanisms of the laminopathies are unclear. The main challenge is to explain how over 500 mutations associated with ubiquitously expressed proteins, give rise to a relatively low number of pathologies (less than twenty) that affect only a limited number of tissues, above all the mechanically stressed muscles [11,25,197]. Following the knowledge that cells expressing mutated A-type lamins present lobulations in the nuclear envelope, loss of peripheral heterochromatin, and anomalous nuclear pore complex distribution, two main models were hypothesized to explain the onset of laminopathies [8,15,183]. According to the “structural model”, the mutation in A-type lamins alters the nuclear resistance to external mechanical stimuli, resulting in nuclear fragility, increased stress sensitivity, and possible premature senescence. This model would explain why the striated muscle tissues, which are the most exposed to mechanical strain, are mainly affected by laminopathies [8,10,197]. The second hypothesis, the “gene expression model”, suggests that altered gene expression is mainly induced either by impaired lamin-chromatin interactions, changes in chromatin organization, or deregulation of the specific genes peripheral position [8,16,99]. In this model, A-type lamin mutations cause gene deregulation, leading to the tissue specificity, which is a feature of most laminopathies [198,199,200]. In fact, some studies show how A-type lamin mutations can alter the gene expression either directly, through their link with heterochromatin, or indirectly by the disruption of protein interactions [74,83,201]. Primarily, it has been observed that loss of A-type lamins was associated with the impaired activation of mechanosensitive genes such as *EGR1* and *IEX1* [202]. Moreover, HGPS fibroblasts have shown heterochromatin loss at the nuclear periphery and altered histone methylation, confirming the deregulation of gene expression due to A-type lamin mutation [100]. Finally, in the case of EDMD, it was also observed that gene-deregulation induced by lamin mutation can affect the stem cell proliferation and differentiation capability. This observation is based on experiments in which EDMD-affected myoblasts still maintain a high proliferative rate, and are no longer able to differentiate [203]. In line with the lamina role in cell mechanotransduction described in Section 5 (Figure 6), it is possible to strike a balance between both these models by focusing on how the lamina acts as both mechanosensor and mechanotransducer [138,179,202,204]. We here report the supporting evidence of impaired mechanotransduction, and its consequences on other cellular mechanisms, in the case of age-related and myopathies diseases.

Several studies have focused on mutations associated with age-related diseases such as Hutchinson Gilford Progeria Syndrome (HGPS) and restrictive dermopathy (RD) and their effects on lamina-impaired structure and activities [205]. In particular, HGPS has attracted much attention because of its similarity to the physiological aging process, both involving the expression of a mutant prelamin A named progerin [206,207]. HGPS is a rare genetic disease, usually caused by a mutation in the exon 11 of *LMNA* gene, as a consequence of the substitution of 1824 nucleotide C for T, which determines the translation to the mutant prelamin A progerin. The progerin protein is characterized by a deletion of C-terminal 50 amino acids including the ZMSPTE24 cleavage site [191,201,208,209]. The permanently attached farnesyl group causes lamin A to accumulate at the inner nuclear membrane leading to extreme lamina stiffness and therefore an impaired mechanotransduction pathway [179,207,210,211]. Moiseeva et al. proposed a self-reinforcing aging mechanism, based on the defective lamin A Ser22 phosphorylation site exhibited in HGPS cells; they suggested that a small accumulation of progerin inhibits the Ser22 phosphorylation site in lamin A, thus preventing Cdk 4/6-kinase activity during interphase. The missed turnover enhances the progerin accumulation leading to the impaired lamina structure [76]. In line with this, the nuclear stiffness in HGPS cells increases with increasing passage in culture. The increased nuclear stiffness in turn alters the lamina sensitivity to external stimuli and therefore its ability to rearrange, according to the different stress conditions [142,201,204,212]. For instance, HGPS skin fibroblasts subjected to 24 h of repetitive biaxial strain, showed larger fractions of propidium iodide-positive cells compared to unstrained and healthy cells, which is an index of the increased mechanosensitivity induced by biomechanical strain [212]. More recently, Bikkul et al. investigated how a Farnesyltransferase inhibitor (FTI) influences genome organization. They demonstrated the combinatorial FTI drug effect in restoring specific chromosome positioning toward the nuclear periphery. The drug probably acts by restoring the ability of unfarnesylated progerin to make the physiological lamina connection, presumably through LADs [213]. As highlighted in Figure 6, altered mechanotransduction may also be caused by cytoskeletal impairment, as reported in a progeria mouse model that showed reduced expression of the cytoskeletal vimentin, a protein not only involved in cell-matrix stabilization via association with integrins, but also in withstanding the mechanical force of cells [209]. Moreover, progerin expression in vascular smooth muscle cells returned impaired LINC complexes at the nuclear envelope due to one of its main proteins named SUN1. The authors showed the stabilization, accumulation, and reduced motility of SUN1, which may cause impaired shear stress sensitivity at the lamina level [214]. In the same context, it has been demonstrated that the reduced force transmission due to LINC disruptions in HGPS-affected smooth muscle cells, ameliorate aortic diseases, resulting in less DNA damage, fewer nuclear blebs, and reduced cell death [204]. In addition, Simon et al. showed that the deletion of C-terminal 50 amino acids in progerin affects one of the two nuclear actin-binding sites. This then leads to impaired rearrangements of nuclear actin, in response to mechanical stresses and contributes to the aberrant structure of the lamina and of the nuclei in HGPS cells [104]. In fact, nuclear actin is essential for nuclear envelope integrity via interactions with lamins and emerin, another A-type lamin binding partner [104,106,215]. Whatever the main cause of the impaired lamina structure and mechanical properties, it affects the translocation of altered nuclear proteins, as reported in physiological aged cells and in both HGPS and RD [216,217,218]. In addition, the pathological lamina alters the chromatin condensation and rearrangement, affecting its accessibility to nuclear proteins [216]. Indeed, progerin accumulation in skin fibroblasts has been correlated to altered repressive histone mark H3K27me3, disrupted heterochromatin-lamina interactions, and loss of natural chromatin compartmentalization between active and inactive zones [219]. According to all this evidence, McCord et al. suggested that progerin accumulation leads to a disruption in the normal nuclear envelope scaffold. This alteration affects the chromatin-lamina association and the heterochromatin distribution of the mark H3K27me3, with reduced LADs in the same gene-poor genomic regions, and loss of spatial chromatin compartmentalization at late cell passages [219]. In fact, both HPGS and RD mutation have been correlated to the loss or rearrangement of heterochromatin and instable genomes, supporting the role of *LMNA* mutation in perturbing the epigenetic control of the chromatin structure [99,211,217,220,221]. Changes in chromatin organization and epigenetic regulation may in turn have a profound impact on gene expression and genome stability [162]. In particular, the reduced level of heterochromatic histone marks has been associated with impaired retinoblastoma protein signaling thus affecting the proliferation of altered stem cells in HGPS [202,222,223]. Moreover, HGPS cells have been correlated to impaired signaling pathways, such as the Notch and Wnt/β-catenin signaling that affect the cell fate regulation-differentiation and ECM-gene expression [90,190,224,225]. Indeed, Notch proteins are transmembrane proteins principally constituted by an extracellular surface receptor and a Notch intracellular domain. The presence of neighboring cells activates the notch-dependent signaling by cleaving the intracellular domain. The cleaved domains translocate to the nucleus and activate the target gene expression. HGPS-related cells showed upregulation of Notch-regulated genes (e.g., *HES1*, *HES5*, *HEY1*, and *TLE1*) caused by reduced levels of the transcriptional corepressor NcoR and the increased nuclear level of the transcriptional coactivator SKIP, which in physiological conditions associates with the nuclear matrix [226]. In line with the Notch-related signaling role in cell fate regulation and stem cell differentiation, Scaffidi et al. found an altered differentiation potential in progerin-expressing cells that exhibited enhanced osteogenesis compared to the adipogenesis [227]. On the other hand, Wnt-proteins are signaling proteins involved in embryonic development and adult cell self-renewal. Once linked to its receptor, a Wnt-protein causes the β-catenin cytoplasmic accumulation. This accumulation in turn causes the β-catenin translocation to the nucleus where it binds to the T-cell factor (TCF)/lymphoid enhancer factor (LEF) thereby inducing the target gene expression [90,226]. Hernandez et al. suggested the inhibition of Wnt signaling as a possible cause of proliferative arrest and death of postnatal fibroblast in a progeric mouse model. They found a reduced nuclear localization and transcriptional activity of LEF1 which, in addition to a defective ECM synthesis role, could represent a critical factor for HGPS etiology [225]. These observations, combined with the increased mechanical sensitivity of HGPS-affected cells, may be the cause of mesenchymal stem cell death and inefficient repair of damaged tissue [202].

Even more evident than in the age-related pathologies, the myopathies that mainly affect the load-bearing tissue, showed altered lamina mechanical response [228]. In this class of diseases, Emery-Dreifuss muscular dystrophy is the first ever studied. Historically the first form of EDMD described in 1961 by Dreifuss and Hogan, later renamed as type 1 (EDMD1), was caused by mutations of Emerin [229]. Then, several *LMNA* gene mutations mainly disseminated in exons 1 and 6 have been associated to EDMD, type 2 and type 3 [230]. Some human *LMNA* EDMD-related mutations in the Ig-fold domain, such as the mutation R453W in human and L535P in *C.Elegans*, have been associated with up to a five-fold reduction in lamin phosphorylation and lower nuclear deformation in muscle cells [82,87,140,228]. This evidence suggests that the impaired Ig-fold domain behavior affects its accessibility to cytokines (e.g., TGF-β1, TGF-β2 and interleukin 17) with a consequent reduced phosphorylation [231,232]. Phosphorylation reduction leads to an increased amount of peripheral lamins and therefore, to a reduced nuclear deformation and higher nuclear resistance to the strain [228]. In contrast, Mitsuhashi et al. reported a particular phosphorylation site (S458) for myopathy-related Ig-fold mutated lamins (R453W) that would seem to increase lamin solubility [139]. In line with Mitsuhashi, another EDMD-related lamin mutation in mouse myoblasts (*LMNA* N195K) was correlated to increased nuclear deformability [183]. Focusing on skeletal muscle dystrophy, Dutta et al. showed an increased oligomerization propensity in the W514R-mutated Ig-fold domain, which manifests itself in the form of a misshapen laminar network and an abnormal distribution of the nuclear pore complexes and, therefore, a defect in nuclear transport [233]. Given all this evidence and although the correlation between the amount of peripheral-lamins and lamin-mutation is not fully understood, the myopathic cells would seem to be characterized by an impaired lamina structure. For instance, Mio et al. found a disruption in the assembly of lamins A, decreased viscosity, and abnormal paracrystal formation in lamin Ig-fold mutated cells [234]. An increase or a decrease in the mechanical properties of lamina cause several downstream consequences, such as improper nuclear deformation and rupture, nucleocytoplasmic translocation of transcription factors, and mechanical force transmission to the chromatin. Indeed, while an increase in peripheral lamins leads to greater nuclear fragility, fewer lamins correspond to a greater mechanical stress per fiber and, therefore, NE rupture [234]. In fact, constant mechanical stress applied to mutant lamina protofilaments causes muscular-specific nuclear rupture, cell death and tissue deterioration [235,236]. For instance, *N195K*-mutated muscular cells show reduced nuclear stability, transient rupture of the NE, chromatin protrusion and DNA damage [183]. On the other hand, the lamina pathological activity affects the translocation of nuclear molecules, as demonstrated in congenital muscular dystrophy, where its impaired sensitivity causes an altered activation of YAP signaling [8,143]. Congenital muscular dystrophy-related cells have been correlated to increased YAP nuclear localization due to increased nuclear import, induced by nuclear envelope defect [237]. Likewise, EDMD mice models show enhanced nuclear translocation of activated extracellular signal-regulated kinase (ERK) and c-Jun N-terminal kinase (JNK). Both ERK and JNK are part of the mitogen-activated protein kinase (MAPK) group, which regulates diverse cellular programs according to extracellular signals. The increased amount of nuclear JNK and ERK, probably induced by abnormal cell responses to stress, enhances the activation of transcription factors such as Elk1, bcl-2, JunD, c-Jun, and Elk4, altering the expression of these genes with a possible impact on cardiomyopathy development [199]. In addition, dilated cardiomyopathy cells show impaired nuclear translocation and downstream signaling of the mechanosensitive transcription factor megakaryoblastic leukaemia 1, which plays a pivotal role in cardiac development [202]. An impaired distribution of lamins influences the altered force transmission to the chromatin and therefore its localization, too. EDMD has been correlated to heterochromatin disorganization with consequences on the genes transcription and improper cellular differentiation [203,236,238,239]. The impaired lamina behavior acts on the transcription active site via LADs chromatin portions which, combined with molecule delocalization in the cell nucleus, enhance the alteration in gene expression [14,74,240]. This alteration may result from mutation-specific altered LADs that modify DNA methylation patterns. In line with this, EDMD-affected cells show overexpression of Sox2 pathway loci, which are involved in cell fate specification and the transcriptional regulation of cell fate commitment. This loss in the heterochromatin formation of Sox2 locus appears to cause maintenance of cellular pluripotency, thus inhibiting and delaying the myogenic differentiation [99]. Mattout et al. found an impaired sequestration of heterochromatin at the NE level and down regulation of at least 24 muscle-specific genes, in LMN-1 Y59C-expressing worms, linked to an autosomal-dominant form of Emery-Dreifuss muscular dystrophy in human [236]. In fact, various studies have reported impaired gene regulation caused by different lamin-related mutations. For instance, while in the presence of *LMNA* R453W-mutated lamins the cells reduced their differentiation activity and showed a low level of expression of the transcription factor myogenin, in the case of C. elegans *LMN-1* L535P lamin (corresponding to human *LMNA* L530P) mutation, the cells showed instead altered regulation of important genes for the pharyngeal muscle [203,238]. This altered gene expression has also been found in congenital muscular dystrophy cells correlated with the impaired expression of mechanosensitive genes *EGR-1* and *IEX-1* [8,143]. This evidence would seem to indicate that the mutation of specific lamins can affect specific chromatin sites inducing specific altered gene transcription. In the same context, EDMD myoblasts cells have been reported to show impaired differentiation activity and also impaired cell polarization and migration speed [99,203,241,242]. It is worth noting that the same laminopathic effects have been observed irrespectively of the deregulated element of the mechanotransduction pathway (e.g., actin filaments, LINC complex, emerin protein) [243]. For instance, atrial cardiac defect-affected myofibroblasts showed reduction of both emerin and SUN2 proteins, supposed to cause the reduced formation of F-actin stress fibers in cyclic stretches condition [244]. Moreover, the same YAP nuclear accumulation previously reported in mutant lamins has been revealed in congenital myopathy with a mutation in nesprin-1 protein [237]. The common element to all the laminopathies would thus appear to be deregulated cell functionality induced by impaired mechanotransduction.

## 6. Conclusions

The lamina is a mesh-like structure that supports the integrity of the nucleus. It mainly consists of lamins, dynamic type V intermediate filament proteins, that supply distinct mechanical properties to the lamina meshwork. The lamina first acts as a mechanosensor able to sense the external stimuli, then, as a mechanotransducer that converts the information into other cellular responses. The overall cellular response is mainly governed by *LMNA* human gene encoded A-type lamins via the nuclear stiffness modulation and the chromatin remodeling. Indeed, acting as a shock absorber, the A-type lamins influence the nuclear influx of transcription factors and modulate the amount of external insults transmitted to the nucleus with consequences on the nuclear damage, nuclear positioning, cell migration, differentiation, and apoptosis. In this context, it was reasonable to suppose that the almost 500 laminopathic mutations mapped to the human *LMNA* gene may be involved in altered protective pathways and impaired transcriptional activation, as suggested by the “structural model” and the “gene expression model” theory, respectively. According to the “structural model”, the mutation in A-type lamins alters the nuclear resistance to external mechanical stimuli, resulting in nuclear fragility, increased stress sensitivity, and possible premature senescence. The second hypothesis, the “gene expression model”, suggests that altered gene expression is mainly induced either by impaired lamin-chromatin interactions, or changes in chromatin organization or deregulation of the peripheral position of specific genes. A-type lamin mutations cause defective lamina rearrangement according to external stimuli that in turn affect the lamina role as shock absorber causing nuclear damage. On the other hand, the impaired force transmission to the lamina induces an impaired chromatin remodeling with consequences on abnormal transcription gene activation involving adaptive and protective pathways.

In this work we have reconciled the two models basing on mechanotransduction mechanisms. To support the mechanotransduction triggering-based mechanisms we reported the most relevant evidence related to Hutchinson-Gilford progeria syndrome and the Emery-Dreifuss muscular dystrophy. Further studies are required to better understand the role of lamins in nuclear processes, aiming to revealing the mechanisms behind human diseases and possible strategies to treat the laminopathies.

## Figures and Tables

**Table 3:** Main binding partners of human lamin A/C.

Binding Partner	Description	A-Type Lamin Binding Region	Reference
Lamin B	Despite their distinct assembly pathways, “the stage is set” for the assembly of A-type lamins.	ND *	[36]
Nuclear Actin	Essential for the integrity of the nuclear envelope, it mediates chromatin movement during transcription and mitosis. Failure of this binding impairs the role of nuclear actin, as happens in Hutchinson-Gilford Progeria Syndrome (see details in Section 5).	461–536 and 564–608	[103,104]
Emerin	Protein of the LEM-domain family; it is relatively immobile in the INM and it anchors the lamina. It also binds directly to the barrier-to-autointegration factor (BAF), retaining chromatin close to the nuclear envelope during cell interphase and acts on gene expression inhibition. Loss of emerin causes Emery-Dreifuss muscular dystrophy (see details in Section 5).	384–566	[105,106,107]
LAP1	Integral membrane protein that binds both A- and B-type lamins. Its role has not been characterized yet, but it is involved in Primary dystonia, a central nervous system laminopathy caused by a mutation in torsin A.	ND *	[108]
LAP2α	The most-studied architectural partner for A-type lamins. It is located inside the nucleus and is necessary to maintain lamin A/C in a soluble and low-assembly state. Its binding to transcriptional regulators suggests its influence in gene regulation either directly, or indirectly through the lamins. Mutations in LAP2α which disrupt the binding to the A lamin are known to cause dilated cardiomyopathy.	319–566	[101,109,110,111]
Nesprin 1α	Nuclear membrane protein that directly binds A-type lamins and emerin and anchors them at the nuclear envelope. Human fibroblasts lacking A-type lamins present mis-localized nesprin 1α and emerin (which are located at the endoplasmic reticulum level) inducing an impaired nuclear geometry and peripheral chromatin loss as occurs in Emery-Dreifuss muscular dystrophy.	ND *	[112,113]
SUN1/2	Essential during cell mitosis. Its important role has been recently suggested in anchoring and opening the nuclear pore complex, and therefore, regulating the nuclear influx of transcription factors.	389–664	[6,7,114,115]
SREBP1 a/c	Known to activate genes required for cholesterol biosynthesis and adipocyte differentiation. They bind the Ig-fold domain of A-type lamins. Deregulation of this binding is involved in lipodystrophies.	389–664	[116,117]
MAN1	LEM-domain protein; it binds BAF directly, but also DNA. It is involved in TGF-β-signaling, important for bone development.	394–664	[118,119]
PKCα	Serine/threonine kinase, activated by many signal pathways and involved in lamin phosphorylation. Once activated, it translocates to the nucleus and binds to the A-type lamin tail to trigger post translational modifications.	500–664	[120]
12(S)-LOX	Lamin binding enzyme 12(S)-lipoxygenase converts arachidonic acid (AA) to 12(S)-hydroxy eicosatetraenoic acid [12(S)-HETE] and is involved in the lipid signaling pathway. It also activates PKCα mediating prostate tumor cell metastasis.	463–664	[121,122]
cFos	Early response transcription factor sequestrated at the nuclear envelope by A-type lamins. During MAP kinase signaling, this binding is released and c-Fos can facilitate cell proliferation.	81–219, 243–388 and 453–571	[123,124]
Rb	Transcriptional regulator that has a central role in cell-cycle control and in apoptosis mechanisms. It directly binds to A-type lamins and to LAP2α. It appears that Rb tumor suppressor activity depends on its attachment to both proteins.	247–355	[125,126]
MOK2	DNA-binding transcriptional repressor that modulates gene expression activated by the cone-rod homeobox protein (Crx), by competing binding to the same binding sites. It also seems to influence RNA processing.	243–387	[127,128]
IMPORTIN α	Nuclear import receptor. It is supposed to prevent lamins from assembling in the nucleoplasm.	ND *	[129]
BAF	Non-specific double-stranded DNA-binding protein. It can bridge DNA and interacts with histones. It also binds several transcription activators including Crx, with an analogous function to MOK2. Alterations in BAF expression lead to impaired chromatin structure, nuclear envelope defects and altered gene expression.	432–544	[105,107,130,131]
LAD	Lamina-associated domains containing lowly transcribed genes. They are dynamic structures involved in chromosomes organization, gene repression, and cell differentiation. LAD disruptions have been correlated to diseases such as Hutchinson Gilford progeria syndrome (see details in Section 4 and Section 5).	ND *	[109,132,133]
Core histones	Their interaction with A-type lamins affects chromatin localization and gene expression.	396–430	[100,102]
PCNA	Necessary to activate the DNA replication machinery, it binds to the Ig-fold domain.	436–552	[134]
DNA	The lamin-DNA interaction occurs directly, but non-specifically, by contacting the minor groove. The DNA-binding region is identical in both lamin A and lamin C. Some lamin A mutations drastically reduce the DNA affinity, leading to gene regulation problems.	411–553	[135,136]

* ND = not determined, yet.

**Table 4:** Classification of laminopathies.

Group	Pathology	OMIM Code	Gene Involved	Reference
1	Emery-Dreifuss muscular dystrophy, autosomal dominant (EDMD2)	181350	*LMNA*	[171,174,183,184,185]
1	Emery-Dreifuss muscular dystrophy, autosomal recessive (EDMD3)	616516	*LMNA*	[174,184,185]
1	Limb-girdle muscular dystrophy, type 1B (LGMD1B)	159001	*LMNA*	[171,174]
1	Congenital muscular dystrophy (CMD)	613205	*LMNA*	[172]
1	Autosomal dominant spinal muscular atrophy (AD-SMA)	182980	*LMNA*	[173]
1	Dilated cardiomyopathy 1A (CMD1A)	115200	*LMNA*	[175,184,186]
1	Dilated cardiomyopathy with conduction system defects (DCM-CD)	n/a	*LMNA*	[187,188]
2	Dunnigan-type familial partial lipodystrophy (FPLD2)	151660	*LMNA*	[171,177,184,189]
2	Metabolic syndrome (MS)	n/a	*LMNA*	[176]
2	Barraquer-Simons syndrome (acquired partial lipodystrophy -APL)	608709	*LMNB2*	[44]
3	Charcot-Marie-Tooth disease, type 2B1 (CMT2B1)	605588	*LMNA*	[178]
3	Autosomal dominant leukodystrophy (ADLD)	169500	*LMNB1*: present an extra copy of the gene	[45]
4	Hutchinson-Gilford progeria syndrome (HGPS)	176670	*LMNA*: LaminA-Δ50 permanently farnesylated	[171,184,190,191,192]
4	Atypical Werner syndrome (WRN)	277700	*LMNA*	[171,180,187]
4	Restrictive dermopathy (RD)	275210	*LMNA*	[193]
4	Mandibuloacral dysplasia with type A lipodystrophy (MADA)	248370	*LMNA*	[171,181,184,194,195]
4	Heart-hand syndrome, Slovenian type (HHS)	610140	*LMNA*	[196]

Laminopathies subdivided in four groups: myopathies (1), lipodystrophies (2), neuropathies (3), and systemic diseases (4). Figure 8 shows all the specific mutations of the *LMNA* gene for each pathology (here omitted for the sake of clarity).
